# Lipase-Produced Hydroxytyrosyl Eicosapentaenoate is an Excellent Antioxidant for the Stabilization of Omega-3 Bulk Oils, Emulsions and Microcapsules

**DOI:** 10.3390/molecules23020275

**Published:** 2018-01-29

**Authors:** Taiwo Olusesan Akanbi, Colin James Barrow

**Affiliations:** Centre for Chemistry and Biotechnology, Deakin University, Locked Bag 20000, Geelong, VIC 3220, Australia; taiwo.akanbi@deakin.edu.au

**Keywords:** hydroxytyrosol, eicosapentaenoic acid, antioxidant, microencapsulation, lipase, *Candida antarctica* B, enzyme, conjugation

## Abstract

In this study, several lipophilic hydroxytyrosyl esters were prepared enzymatically using immobilized lipase from *Candida antarctica* B. Oxidation tests showed that these conjugates are excellent antioxidants in lipid-based matrices, with hydroxytyrosyl eicosapentaenoate showing the highest antioxidant activity. Hydroxytyrosyl eicosapentaenoate effectively stabilized bulk fish oil, fish-oil-in-water emulsions and microencapsulated fish oil. The stabilizing effect of this antioxidant may either be because it orients itself with the omega-3 fatty acids in the oil, thereby protecting them against oxidation, or because this unstable fatty acid can preferentially oxidise, thus providing an additional mechanism of antioxidant protection. Hydroxytyrosyl eicosapentaenoate itself was stable for one year when stored at −20 °C.

## 1. Introduction

Olive oil phenols, particularly hydroxytyrosol and tyrosol, are natural polyphenols with antioxidant activity and known health benefits [1]. Hydroxytyrosol has been demonstrated in numerous studies to have cardioprotective properties, by preventing oxidative damage to low-density lipoproteins (LDLs), and as a result inhibits the activation of inflammatory events and prevents endothelial damage, the major causes of atherosclerosis [2,3]. It was shown to diminish the secretion of inflammatory cytokines (IL-1α, IL-1β, IL-6, IL-2, TNF-α) and chemokines (CXCL10/IP-10, CCL2/MCP-1) [3]. The European Food Safety Authority (EFSA) has concluded that a cause-and-effect relationship exists between the consumption of hydroxytyrosol and protection against oxidative damage of blood lipoproteins [4]. Hydroxytyrosol is currently marketed as a nutritional supplement in several countries.

The use of synthetic antioxidants in food and other nutritional products is not accepted by consumers and regulators in some countries, shifting the attention of manufacturers to natural antioxidants such as polyphenols. However, there are a limited number of natural antioxidants that can be incorporated into lipid-based food matrices [5]. The hydrophilic property of hydroxytyrosol limits its dispersion in fats and oils, thereby reducing its effectiveness in protecting lipids against oxidation. Lipophilic derivatives of hydroxytyrosol will have improved oil solubility and antioxidant properties in fats and oils. Esterification of hydroxytyrosol with fatty acids has been carried out chemically and enzymatically [5,6,7]. Lipase-catalysed esterification reactions can be carried out under mild conditions and with less side reactions compared with non-enzymatic methods [8,9].

Previous studies have found that an increase in the chain length of fatty esters of hydroxytyrosol, up to a certain chain length, correlated with an increase in antioxidant activity [5,6,7,10,11]. However, the majority of these studies focused on the antioxidant activity of short to medium chain alkyl esters of hydroxytyrosol. Recent studies have shown significantly higher antioxidant activity of the unsaturated fatty esters of polyphenols over their saturated counterparts [12,13], indicating that chain length and the degree of unsaturation of fatty acids has an impact on the antioxidant activity of polyphenol esters. For instance, it has been shown that the antioxidant activity of polyunsaturated fatty acid derivatives of quercetin (Q3G) increased with an increasing number of double bonds [13]. Although the omega-3 esters of hydroxytyrosol have been previously synthesized [14,15], their abilities to stabilize omega-3 oils were not explored.

A combination of different testing systems is needed to ensure a comprehensive assessment of an antioxidant. Studies have shown that the physical nature of lipid medium has a significant impact on the efficacy of antioxidant [16,17]. It has also been shown that the effectiveness of some fatty acid derivatives of polyphenols differ in bulk oil and in oil-in-water emulsions [17]. While the antioxidant activities of phenolic esters have been widely reported, studies on their stability over extended period of storage are lacking. The ability of hydroxytyrosyl esters to stabilize microencapsulated fish oil has not been previously investigated. Therefore, in this study, we enzymatically synthesized and purified lipophilic derivatives of hydroxytyrosol and tested their antioxidant activities in a range of synthetic and lipid-based matrices, including bulk fish oil, fish oil-in-water-emulsion and microencapsulated fish oil. The stability of these antioxidants over an extended period of storage was also investigated.

## 2. Results and Discussion

### 2.1. Lipase-Catalysed Synthesis of Hydroxytyrosol-Fatty Acid Conjugates

Esterification conditions were optimized for the synthesis of hydroxytyrosyl decanoate using immobilized lipase from *Candida antarctica* B (Novozym 435) and were applied to the synthesis of all conjugates. These conditions were: Solvent (diethyl ether), temperature (40 °C), molar ratio of substrates (hydroxytyrosol-fatty acid ratios; 1:2) and reaction times (6 h). Other general conditions are as described in the methods section. Immobilized lipase was used in preference to free lipase since the immobilized form tends to be more robust and is more widely used at the manufacturing scale due to its ease of recovery and reuse.

### 2.2. Characterisation of Hydroxytyrosyl Esters

The synthesised hydroxytyrosyl esters are shown in Table 1. As shown, the shorter the fatty acid chain, the higher the conversion rate of hydroxytyrosol. All saturated fatty acids except stearic acid gave higher percent conversion than unsaturated fatty acids. This may be due to the straight chain-like structure of saturated fatty acids, which allows them to stack tightly and have more intermolecular interactions [12]. However, with the long chain unsaturated fatty acids, there appears not to be a relationship between chain length and conversion, as higher conversion was obtained with eicosapentaenoic acid (EPA) (68%) than with oleic acid (46%). Although some studies have shown that conjugation of longer chain fatty acids with polyphenols results in low yield [12,18], others have shown the opposite [5,19]. The variation in results may be due to lipase fatty acid selectivity primarily differentiating degree of unsaturation rather than fatty acid chain length.

**Figure 1:**
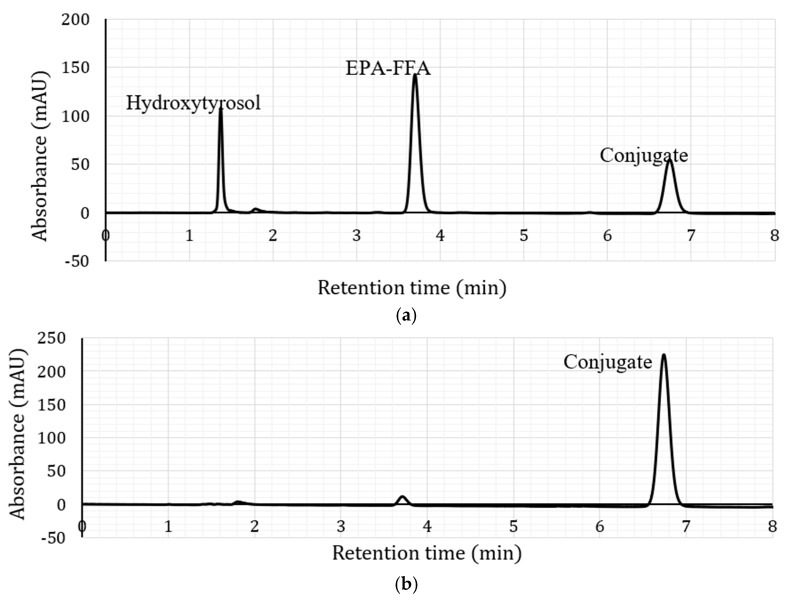

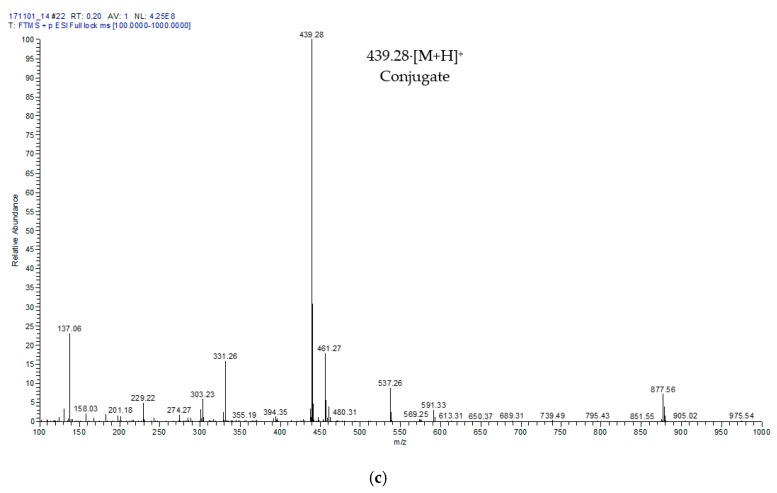
HPLC chromatograms of hydroxytyrosyl eicosapentaenoate (**a**) before and (**b**) after purification and its (**c**) LCMS spectrum.

The results of the liquid chromatography-mass spectrometry (LC-MS) and proton-nuclear magnetic resonance (^1^H-NMR) analyses of the conjugates are shown in Table 1. The high-performance liquid chromatography HPLC chromatogram and LC-MS spectrum of a representative sample (hydroxytyrosyl eicosapentaenoate) are shown in Figure 1. Results (Table 1 and Figure 1) show that the conjugates were synthesized as anticipated, since they had the expected mass/charge ratios (*m*/*z*). For ^1^H-NMR, the chemical shift assignments (δ, ppm) for free hydroxytyrosol were: δ 3.69 (2H, t, H-1′), 2.66 (2H, t, H-2′), 6.66 (1H, d, H-4′), 6.70 (1H, d, H-7′) and 6.54 (1H, dd, H-8′). Selected ^1^H-NMR results are presented in Table 1. As shown, no allylic, olefinic or bis-allylic protons were detected in all the saturate-based conjugates, whereas bis-allylic protons were found in hydroxytyrosyl eicosapentaenoate but not in hydroxytyrosyl oleate, consistent with the predicted structures.

### 2.3. Antioxidant Activity by Spectrophotometric Methods

#### 2.3.1. DPPH Radical Scavenging Activity

The 2,2-diphenyl-1-picrylhydrazyl DPPH radical scavenging assay is a rapid, substrate-free technique for determining antioxidant activity. The results of the DPPH radical scavenging assay of conjugates are expressed as EC_50_, that is, the concentration necessary to lower the initial DPPH by 50%, and are presented in Table 2. As shown, there are no significant differences (*p* > 0.05) between the values obtained for hydroxytyrosol and the conjugates, indicating that hydroxytyrosol and its lipid derivatives show effective DPPH radical scavenging activity. A similar result was also obtained for butylated hydroxytoluene (BHT), but *α*-tocopherol showed a significantly lower (*p* < 0.05) activity, requiring 9.87 ± 1.03 µM to lower the initial DPPH by 50%. Previous studies have shown that *α*-tocopherol is less active against DPPH free radicals than polyphenols and their acyl esters, with some showing two to three times more scavenging activities than *α*-tocopherol [6,20].

#### 2.3.2. ABTS Radical Scavenging Activity

The 2,2-Azino-bis-3-ethylbenzothiazoline-6-sulphonic (ABTS) radical scavenging test is based on the inhibition of ABTS radicals, which are measured spectrophotometrically in the near-infrared region at 734 nm [21]. Results presented in Table 2 show that all conjugates and hydroxytyrosol exhibit lower EC_50_ values compared to BHT and *α*-tocopherol. Thus, based on concentration, hydroxytyrosol and its acyl esters are more potent antioxidants than BHT and *α*-tocopherol.

The results of both the DPPH and ABTS tests show that the nature of the fatty acyl side chain does not influence the antioxidant activity of the hydroxytyrosol conjugates. However, these results may not reflect the antioxidant activity of these conjugates in real food systems, where the lifetime of radicals are shorter than those of the artificial DPPH and ABTS radicals [18,22]. Since it has been shown that the lipophilicity of polyphenols often enhances their antioxidant activity in certain systems [12,23], further tests are needed to obtain a better understanding of the antioxidant activities of these conjugates in different lipid systems.

#### 2.3.3. Antioxidant Activity in a *β*-Carotene-Linoleate Model System

This method measures the rate of oxidative destruction of *β*-carotene by the free radicals of oxidised linoleic acid in an emulsion system. In the presence of an antioxidant, bleaching of *β*-carotene occurs. In this study, the antioxidant activity of conjugates, BHT and *α*-tocopherol are expressed as percent inhibition relative to the control and results are presented in Table 2. As shown, all the conjugates except hydroxytyrosyl decanoate inhibited the bleaching of *β*-carotene by up to 60%. Hydroxytyrosyl eicosapentaenoate in this system had the highest antioxidant activity of all the conjugates. Although there is no report on the antioxidant activity of hydroxytyrosyl eicosapentaenoate in an emulsion system, studies have shown that some phenolic conjugates of unsaturated fatty acids exhibit antioxidant activities in an emulsion system [12,13]. For instance, unsaturated derivatives of quercetin-3-*O*-glucoside (oleate, linoleate, eicosapentaenoate and docosahexaenoate) have shown significantly higher antioxidant activities than quercetin-3-*O*-glucoside (Q3G) in fish-oil-in-water emulsion [13]. Tyrosyl oleate has also been reported to better stabilize omega-3 rich oil-in-water emulsion than tyrosyl stearate and tyrosol [12].

Hydroxytyrosol itself performed worse than all the test compounds, including BHT and *α*-tocopherol, in this antioxidant assay (Table 2). The behavior of hydroxytyrosol is consistent with the “polar paradox” theory, which postulates that lipophilic antioxidants are more effective in oil-in-water emulsions, while hydrophilic ones are more effective in bulk oil [16]. This explains why the synthesized conjugates with improved lipophilicity showed a higher inhibitory effect on *β*-carotene bleaching than did hydroxytyrosol. Several studies have shown that lyophilized polyphenols are more effective than their parent polyphenols in oil-in-water emulsion systems [12,13,18].

### 2.4. Antioxidant Activity Under Rancimat Conditions

**Figure 2:**
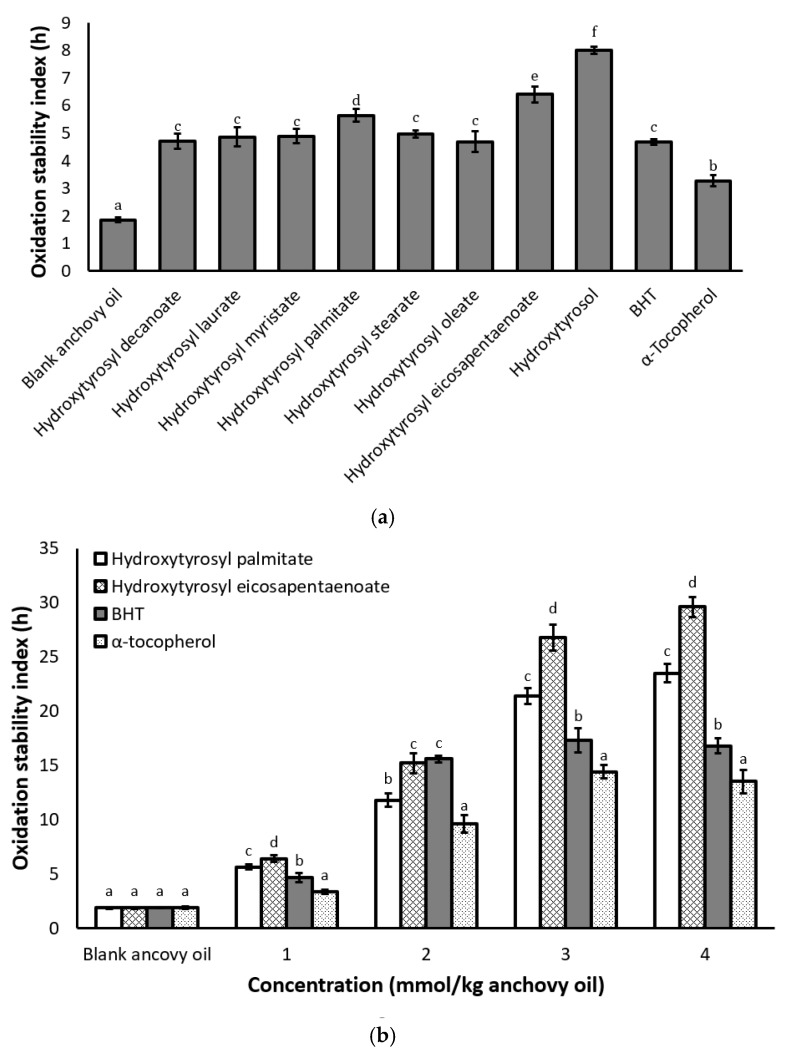
Effects of conjugates, butylated hydroxytoluene (BHT and *α*-tocopherol on the oxidation stability of anchovy oil (**a**) at a concentration of 1 mmol/kg oil and (**b**) at different concentrations. Values are expressed as mean ± standard deviation (*n* = 3). Bars not sharing a common letter are significantly different (*p* < 0.05) within each treatment as determined by Tukey–Kramer HSD.

The antioxidant activities of conjugates were evaluated in anchovy oil at a concentration of 1 mmol/kg oil using the Rancimat accelerated oxidation test method. BHT and *α*-tocopherol were used as standard antioxidants and a temperature of 80 °C was applied to minimize the loss of BHT by evaporation. As shown (Figure 2a), hydroxytyrosol gave the highest oxidative stability index (OSI) value of all the test compounds. This is consistent with the “polar paradox” theory that hydrophilic antioxidants are more effective in bulk oil than lipophilic ones [16]. The partially oil-soluble hydroxytyrosol may be preferentially located at the oil interface and is hence more effective in inhibiting oxidation than the conjugates that are dissolved in the lipid phase. Similar behavior has been reported for trolox, ascorbic, gallic, caffeic and ferulic acids. They showed higher antioxidant activities than their non-polar alkyl esters in bulk oil [17]. Among the conjugates tested, hydroxytyrosyl eicosapentaenoate gave the highest stability, followed by hydroxytyrosyl palmitate, which showed a slightly higher stability than other conjugates (Figure 2a). While it has been reported that short-to-medium-chain lipophilic esters of polyphenols are more effective antioxidants than long-chain esters in bulk oil [17], some studies have shown equal or higher activity with long-chain esters [7,13]. Therefore, the antioxidant activity of lipophilic derivatives of polyphenols is not directly related to the fatty acid chain length.

Since antioxidant activity is concentration-dependent and some antioxidants can exhibit prooxidant behavior at high concentrations, we investigated the effect of concentration on the antioxidant activity of hydroxytyrosyl eicosapentaenoate, palmitate, BHT and *α*-tocopherol in anchovy oil. The results presented in Figure 2b show that increasing the concentration of all test compounds resulted in increased oil stability. However, beyond 2 mmol/kg oil, no further increase was observed for BHT and *α*-tocopherol. Meanwhile, for the hydroxytyrosyl esters, oil stability increased up to a concentration of 3 mmol/kg oil but only increased slightly at concentrations above that. The significantly high stability observed with hydroxytyrosyl eicosapentaenoate over the palmitate (*p* < 0.05) may either be because it orients itself with the omega-3 fatty acids in anchovy oil, thereby protecting them against oxidation, or because this unstable fatty acid can preferentially oxidise providing an additional mechanism of antioxidant protection. A recent study has shown that the antioxidant activity of polyunsaturated fatty acid derivatives of quercetin (Q3G) increased with increasing double bonds [13]. The authors found that at a concentration of 0.5 mmol/L of oil, the inhibition of secondary lipid oxidation (TBARS) in bulk fish oil were 34% (Q3G linoleate), 38% (Q3G linolenate), 46% (Q3G eicosapentaenoate) and 50% (Q3G docosahexaenoate), respectively. The use of synthetic antioxidants such as BHT has been linked to health issues such as liver damage and carcinogenesis [24]. Also, high levels of *α*-tocopherol in oil can result in prooxidation and therefore limits the usefulness of this natural antioxidant when used on its own [25]. It is important to identify new natural antioxidants that can be used alone or in combination with compounds such as *α*-tocopherol to improve the stability of unstable ingredients such as fish oil. Although, hydroxytyrosyl eicosapentaenoate and palmitate showed good antioxidant activity in anchovy oil under Rancimat conditions, further tests are needed to obtain a better understanding of their antioxidant activity in different lipid matrices.

### 2.5. Antioxidant Activity under Schaal Oven Conditions

The effect of hydroxytyrosyl eicosapentaenoate and hydroxytyrosyl palmitate (3 mmol/kg oil) on the oxidative stability of bulk anchovy oil and its oil-in-water emulsion was determined under Schaal oven conditions at 60 °C for five days. Oxidized oil from the oil-in-water emulsion was extracted using methanol:heptane mix (1:1) as previously described [26,27]. Level of oxidation was monitored by applying conjugated diene (CD) and thiobarbituric acid reactive substances (TBARS) tests. CD is an indicator of the formation of primary oxidation products such as hydroperoxides, whereas TBARS are used to determine the presence of secondary oxidation products such as aldehydes and dialdehydes in oils [8,28].

**Figure 3:**
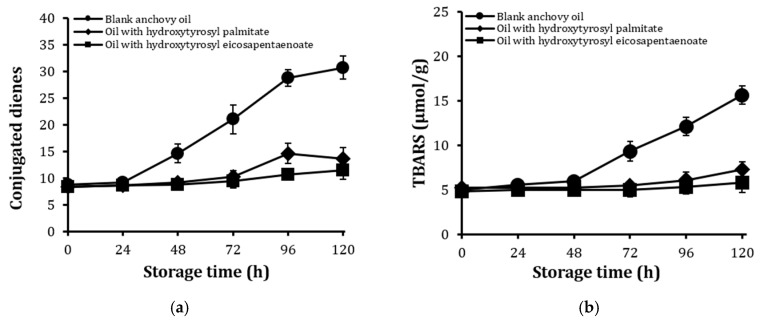

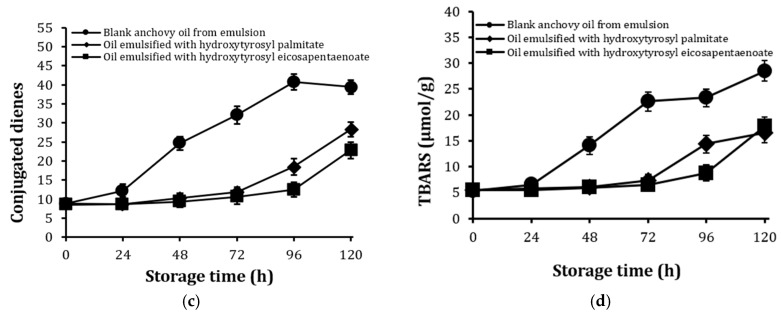
Conjugated dienes (CD) and thiobarbituric acid reactive substances (TBARS) values of bulk anchovy oil (**a**,**b**) and those of the oil extracted from the oil-in-water emulsion (**c**,**d**). Values are expressed as mean ± standard deviation (*n* = 3).

The impact of hydroxytyrosyl eicosapentaenoate and hydroxytyrosyl palmitate on the formation of CD and TBARS in bulk anchovy oil during extended storage is shown in Figure 3a,b, respectively. The conjugates significantly inhibited the release of CD and TBARS throughout the 120 h of storage compared to the control. Their abilities to suppress the formation of CD and TBARS may be attributed to their higher radical scavenging activities [29]. The CD and TBARS values in the anchovy oil emulsion are presented in Figure 3c,d. As shown, both conjugates inhibited CD and TBARS formation up to 72 h, but the values of these oxidation products rose afterwards. This later increase in oxidation may partly be due to the high level of water in the emulsion, compared to that in the bulk oil, which was relatively stable.

Eicosapentaenoic acid (EPA) and docosahexaenoic acid (DHA) derivatives of quercetin-3-*O*-glucoside (Q3G eicosapentaenoate and Q3G docosahexaenoate) significantly inhibited lipid peroxidation in fish oil-in-water emulsion compared with quercetin-3-*O*-glucoside (Q3G) [13]. Increased concentration of Q3G eicosapentaenoate and Q3G docosahexaenoate led to an increase in the inhibition of peroxides and TBARS formation. A 10 mmol/L concentration of Q3G docosahexaenoate in fish-oil-in-water emulsion led to 100% inhibition of peroxidation [13]. These results are consistent with our observations that an increase in the concentration of hydroxytyrosyl esters (hydroxytyrosyl palmitate and hydroxytyrosyl eicosapentaenoate) resulted in increased oil stability. Although the inhibitory effect of hydroxytyrosyl eicosapentaenoate on CD and TBARS formation in bulk oil and oil-in-water emulsion was slightly higher than that of hydroxytyrosyl palmitate, both conjugates are potent antioxidants.

### 2.6. Stability Against Oxidation of Encapsulated Anchovy Oil

**Figure 4:**
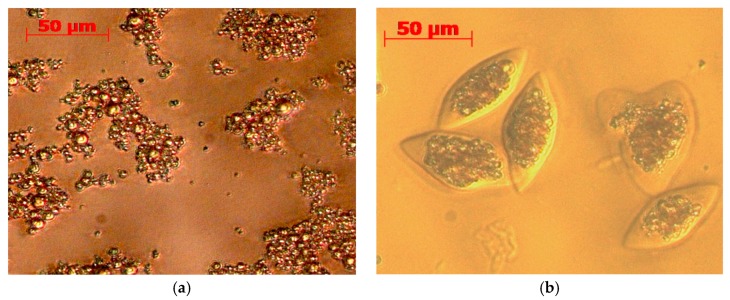

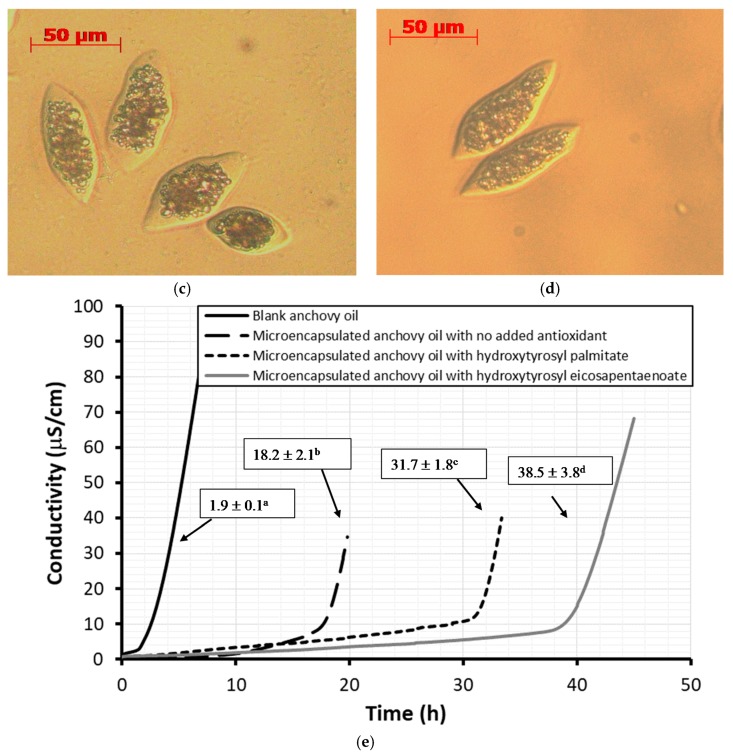
(**a**) The morphology of formed coacervates (blank anchovy oil) in the aqueous phase after agglomeration and microcapsules of (**b**) blank anchovy oil and oil with (**c**) hydroxytyrosyl palmitate and (**d**) hydroxytyrosyl eicosapentaenoate; (**e**) Oxidative stability index of blank and microencapsulated anchovy oil by accelerated oxidation test using Rancimat. Values are expressed as mean ± standard deviation (*n* = 3). Mean values not sharing a common letter are significantly different (*p* < 0.05) as determined by Tukey–Kramer HSD.

Microencapsulation helps protect oil against oxidation, particularly in the presence of antioxidants. We have previously microencapsulated fish oil with gelatin-sodium hexametaphosphate (SHMP) using a multicore complex coacervation technique [30,31]. In this study, we used complex coacervation to microencapsulate anchovy oil with hydroxytyrosyl palmitate and hydroxytyrosyl eicosapentaenoate (3 mmol/kg oil) and tested their stability against oxidation. The morphology of the coacervates in the aqueous phase after agglomeration are presented in Figure 4a, while the microcapsules of blank anchovy oil and oil with hydroxytyrosyl palmitate and hydroxytyrosyl eicosapentaenoate are presented in Figure 4b–d, respectively. For each sample, we obtained a high encapsulation efficiency (between 96% and 98%) and yield (between 94% and 97%) [30,31].

The accelerated oxidative stability tests (Rancimat) of microencapsulated samples are presented in Figure 4e. As shown, the OSI value of blank non-encapsulated anchovy oil (1.9 ± 0.1 h) significantly increased after microencapsulation (18.2 ± 2.1 h) even without an added antioxidant. Much higher increases were observed in the presence of conjugates, with hydroxytyrosyl eicosapentaenoate giving a higher OSI value (38.5 ± 3.8 h) than hydroxytyrosyl palmitate (31.7 ± 1.8 h). These results indicate that lipophilic esters of polyphenols, and in particular hydroxytyrosyl eicosapentaenoate are useful antioxidants for stabilising microencapsulated omega-3 oils.

Microencapsulated fish oil is a functional food ingredient with health benefits associated with the EPA and DHA fatty acids. In addition to acting as an antioxidant, hydroxytyrosyl eicosapentaenoate may also provide some additional health benefits. These may be beyond the known benefits of hydroxytyrosol as an anti-inflammatory compound due to the synergistic benefits of the conjugated EPA, particularly in neurodegenerative diseases [32]. However, exploring the additional health benefits of this conjugate is outside the scope of the current study.

### 2.7. Stability of Conjugates After Extended Storage

The antioxidant activity of hydroxytyrosyl palmitate and hydroxytyrosyl eicosapentaenoate were tested after one year storage in a −20 °C freezer. The results of DPPH and ABTS assays were consistent with those obtained earlier (Table 2), although with slight differences. These were, for hydroxytyrosyl palmitate (DPPH EC_50_, 7.08 ± 0.55 μM and ABTS EC_50_, 2.75 ± 0.68 μM) and hydroxytyrosyl eicosapentaenoate (DPPH EC_50_, 6.18 ± 0.82 μM and ABTS EC_50_, 3.01 ± 0.45 μM). These results show that fatty acid esters of hydroxytyrosol can be stored for use as antioxidants.

## 3. Materials and Methods

### 3.1. Materials

Hydroxytyrosol was purchased from Biotain Pharma Co., Ltd. (Xiamen, Fujian, China) and its purity was confirmed by HPLC, NMR and LC-MS. Anchovy oil was provided by Lipa Pharmaceuticals Pty. Ltd. (Minto, NSW, Australia). Transglutaminase (Activa^®^ KS–LS) was purchased from Ajinomoto (Tokyo, Japan). Gelatin, sodium hexametaphosphate (SHMP), 2,2-Azino-bis-3-ethylbenzothiazoline-6-sulphonic (ABTS), alpha-tocopherol, butylated hydroxytoluene (BHT), 2,2-Diphenyl-1-picrylhydrazyl (DPPH), beta-carotene, immobilized lipase from *Candida antarctica* B, fatty acids, hydroxytyrosol standard and gas chromatography standards were purchased from Sigma Aldrich (Castle Hill, Australia). EPA-ethyl ester (EPA-EE) was provided by Photonz Corporation (Auckland, New Zealand) and was converted to free fatty acid (FFA) as previously reported [8]. Thin layer chromatography standards were purchased from Nu-Chek Prep (Elysian, MN, USA). All other chemicals used were of analytical grade.

### 3.2. Enzymatic Synthesis of Hydroxytyrosyl Esters

Synthesis was first carried out using decanoic acid and hydroxytyrosol to standardize esterification conditions. Reaction was performed in a stoppered bottle consisting 20 mL 2-methyl-2-butanol (2M2B), 2 mmol hydroxytyrosol and fatty acid (1:1), 150 mg immobilized lipase from *Candida antarctica* B and 150 mg molecular sieves (3 Å, 4–8 mesh). The reaction mixture was incubated at 50 °C for 24 h with magnetic stirring at 250 rpm. The progress of the reaction was monitored by capillary chromatography with flame ionization detector (Iatroscan MK5, Iatron Laboratories Inc., Tokyo, Japan). The Iatroscan-FID settings and solvent systems were as previously described [33]. Percentage conversion was calculated as the area of ester peak divided by the combined areas of ester peak and hydroxytyrosol peak.

#### 3.2.1. Optimizing Synthesis Conditions

The effects of different solvents (2-methyl-2-butanol, tert-butanol, isooctane, diethyl ether, ethyl acetate), temperatures (40 and 50 °C), molar ratio of substrates (hydroxytyrosol-fatty acid ratios; 1:1, 1:2, 1:3, 1:5) and reaction times (1–24 h) on the ester formation were investigated by varying one factor at a time while keeping the others constant. The conditions established for the optimum synthesis of hydroxytyrosyl decanoate were then applied for the synthesis of other esters.

#### 3.2.2. Scaled-up Synthesis of Hydroxytyrosyl Esters

Having established the optimum conditions for the synthesis of hydroxytyrosyl esters (including solvent type, temperature, molar ratio of hydroxytyrosol and fatty acid, and reaction time), esterification reactions were scaled up to provide sufficient samples for subsequent experiments. The scaled-up conditions were as follows: 15 mmol hydroxytyrosol and fatty acid (optimized mole ratio), 150 mL solvent, 600 mg immobilized lipase from *Candida antarctica* B and 450 mg molecular sieves (3 Å, 4–8 mesh). The reaction mixture was incubated with magnetic stirring (250 rpm) at predetermined temperature and time.

### 3.3. Identification, Purification and Characterisation of Hydroxytyrosyl Esters

Immobilized enzyme and molecular sieves were removed by centrifugation at 5432× *g* for 5 min and the solvent from the reaction mixture was dried *in vacuo*. The resulting precipitate was resolved in deionized water and heptane (1:5 *v*/*v*) after sparging with nitrogen. Mixture was shaken for 2 min and allowed to stand for another 5 min. Hydroxytyrosyl ester and unreacted free fatty acid went into the organic phase while the unreacted hydroxytyrosol dissolved in the water phase. Silica gel column was then used to separate hydroxytyrosyl ester from the free fatty acid by eluting with heptane/diethyl ether/acetic acid mixtures (50:40:2). The fractions collected were analysed by capillary chromatography with flame ionization detector (Iatroscan-FID) to confirm separation [34]. Samples were also analysed by Agilent 1200 Series HPLC system with the Agilent 1200 Series photodiode array detector using a C18 reversed-phase column, 4.6 mm × 250 mm (Phenomenex, Torrance, CA, USA). HPLC conditions were: mobile phase 90/10 (*v*/*v*), methanol/water, flow rate 1 mL/min, temperature 27 °C, injection volume 10 μL, detection UV detector at 210 nm. Prior to analysis, the column was equilibrated with mobile the phase under the above conditions for 30 min. Pure reactants (fatty acids and hydroxytyrosol) were used as reference standards. ^1^H NMR spectra were acquired on a Bruker Avance 500 MHz spectrometer (Bruker Karlsruhe, Germany) as previously reported [8]. To record ^1^H NMR spectrum, 10 mg of sample was dissolved in deuterated methanol (CD_3_OD; 0.4 mL). Chemical shifts were referenced to the residual solvent peak. Mass spectrometry (MS) spectra of samples were recorded on a Thermo NanoLC/OrbiTrap Fusion Lumos mass spectrometer (ThermoFisher Scientific) using a combination of acetonitrile (90%) and water (10%) as mobile phase.

### 3.4. Measurement of Antioxidant Activity by Spectrophotometric Methods

#### 3.4.1. DPPH Radical Scavenging Method

The DPPH radical scavenging assay was carried out following a previously reported method [35] with some modifications. Briefly, 2 mL of DPPH (40 μM in methanol) solution was added to 50 μL of various concentrations (0, 0.5, 1, 5, 10 and 20 μM) of hydroxytyrosyl esters dissolved in methanol. Contents were thoroughly mixed, incubated at room temperature for 30 min in the dark and absorbance was read at 517 nm in a Cary 300 Bio UV-visible Spectrophotometer (Agilent Technologies). DPPH radical scavenging activity was calculated as:

Radical scavenging activity (%) = [(*A_control_* − *A_sample_*)/*A_control_*] × 100 (1)

where *A_control_* and *A_sample_* are absorbance of control and sample, respectively. Control contained no test compound.

EC_50_ value, which corresponds to the ester concentration necessary to lower the initial DPPH by 50% was determined by linear regression analysis of the graph of scavenging activity (%) vs. concentration. Low EC_50_ value indicates the effectiveness of the antioxidant to act as DPPH scavenger.

#### 3.4.2. ABTS Radical Scavenging Method

The ABTS radical scavenging activity was measured as previously described [36] with minor modifications. ABTS radical cation (ABTS^+^) was generated by reacting 7 mM aqueous solution of ABTS with 2.45 mM potassium persulphate (K_2_S_2_O_8_) and the mixture stood for 16 h in the dark before use. Afterwards, solution was diluted with ethanol to an absorbance of approximately 0.7 at 734 nm (Cary 300 Bio UV-visible Spectrophotometer, Agilent Technologies). Different concentrations (0, 0.5, 1, 5, 10 and 20 μM) of hydroxytyrosyl esters (2 mL) was mixed with the ethanolic ABTS^+^ solution (2 mL) and absorbance was read at 734 nm after 15 min. Scavenging activity and EC_50_ was calculated as described in Section 3.4.1.

#### 3.4.3. *β*-Carotene Bleaching Method

The antioxidant activities of hydroxytyrosyl esters were determined using the *β*-carotene bleaching method [37]. Briefly, *β*-carotene (10 mg) was dissolved in chloroform (10 mL) and an aliquot (1 mL) of this was transferred into a round bottom flask containing linoleic acid (20 mg) and Tween 40 (200 mg). Chloroform was removed *in vacuo* and aerated distilled water (50 mL) was added. Contents were mixed using a magnetic stirrer and 4.8 mL of the mixture was added to test tubes containing 0.2 mL of hydroxytyrosyl esters (1 mM in ethanol) before incubation at 50 °C. Absorbance was measured at 450 nm every 20 min for 120 min. Blank sample was prepared without *β*-carotene and control was prepared without test compounds. Percent inhibition of *β*-carotene-linoleic acid oxidation was calculated as:

(2) $$\mathrm{Inhibition}\;(\%)=100\;\times \left[1-(A_{s0}-A_{s120})/\left(A_{c0}-A_{c120}\right)\right]$$

where A_s0_ − A_s120_ are absorbance values for test samples at 0 min and 120 min while A_c0_ − A_c120_ are absorbance values for control at 0 min and 120 min, respectively.

### 3.5. Accelerated Antioxidant Tests

#### 3.5.1. Preparation of Bulk Oil and Oil-in-Water Emulsion Containing Hydroxytyrosyl Esters

For bulk oil preparation, required amount of hydroxytyrosyl esters were first dissolved in ethanol before being mixed with oil (5 g). Ethanol was then removed *in vacuo*. Oil-in-water emulsion (30 mL) was prepared by emulsifying 3 g anchovy oil, 300 mg Tween 40, required amount of hydroxytyrosyl esters and distilled water. Mixtures were emulsified at 15,000 rpm for 15 min using a homogenizer (Unidrive 1000, CAT Scientific, Paso Robles, CA, USA).

#### 3.5.2. Measurement of Antioxidant Activity Using the Schaal Oven Test

The oxidative stability of bulk anchovy oil and its oil-in-water emulsion was determined under Schaal oven conditions at 60 °C for five days [28]. Briefly, bulk oil (5 g) and oil-in-water emulsion (30 mL) samples (in dark screw-capped bottles) were placed in the oven and samples were withdrawn every 24 h for 120 h (five days). Oxidized oil from the oil-in-water emulsion was extracted using methanol:heptane mix (1:1) as previously described [26,27]. The conjugated dienes (CD) and thiobarbituric acid reactive substances (TBARS) values of the oil samples were measured as previously reported [28].

#### 3.5.3. Measurement of Antioxidant Activity by Rancimat Method

Anchovy oil (4 g) or microencapsulated products (1.5 g) were subjected to accelerated oxidation by Rancimat (model 743, Metrohm, Switzerland) as previously reported [30]. Samples were heated at 80 °C under an air flow rate of 20 L/h and induction time (IT) was recorded for each sample as a measure of their oxidative stability index (OSI).

### 3.6. Microencapsulation by Coacervation

Oil samples (20 g) containing known amounts of hydroxytyrosyl esters were microencapsulated and characterised as previously reported [30]. The formation of complex coacervates was monitored by an Axiotron microscope equipped with a camera (Carl Zeiss GMBH, Oberkochen, Baden-Württemberg, Germany). The encapsulation efficiency (EE), payload (PL) and encapsulation yield (EY) were determined as previously described [30] and oxidative stability tests of microencapsulated products were carried out immediately after freeze drying and milling.

### 3.7. Statistical Analysis

Experiments were carried out in triplicate and data are presented as the mean ± standard deviation (SD). Levene’s test of one-way analysis of variance (ANOVA) was used to verify the homogeneity of variance. Multiple comparisons were achieved by Tukey–Kramer HSD (honestly significant difference). Mean differences were significant when probability is less than 0.05 (*p* < 0.05). Statistical analysis was performed using SPSS^®^ statistics 22.0 software.

## 4. Conclusions

Lipase-catalysed conjugation of hydroxytyrosol with a range of fatty acids was carried out, followed by identification and purification of the resultant conjugates using Iatroscan-FID, mass spectrometry (MS) and nuclear magnetic resonance (NMR) spectroscopy. All conjugates exhibited high scavenging activity towards 2,2-Diphenyl-1-picrylhydrazyl (DPPH) and 2,2-Azino-bis-3-ethylbenzothiazoline-6-sulphonic (ABTS) radicals, with hydroxytyrosyl eicosapentaenoate exhibiting the strongest inhibitory effect *β*-carotene bleaching. In bulk oil, hydroxytyrosyl eicosapentaenoate resulted in the highest stability, followed by hydroxytyrosyl palmitate, with both showing higher stabilising activity than BHT and *α*-tocopherol. Hydroxytyrosyl eicosapentaenoate also showed superiority in stabilizing fish-oil-in-water emulsion, as determined through CD and TBARS tests during accelerated oxidation at 60 °C for five days. The effectiveness of these antioxidants, particularly hydroxytyrosyl eicosapentaenoate, for stabilizing microencapsulated fish oil was also demonstrated. These results indicate that lipase synthesized esters of hydroxytyrosol, particularly hydroxytyrosyl palmitate and hydroxytyrosyl eicosapentaenoate, are useful antioxidants for stabilizing omega-3 oils such as fish oil and for addition to oil/fat-based foods. Further studies are needed to investigate if these conjugates have similar or different bioactive properties to hydroxytyrosol.

## Figures and Tables

**Table 1:** Identification and characterization of conjugates by liquid chromatography-mass spectrometry (LC-MS) and proton-nuclear magnetic resonance (^1^H-NMR) spectroscopy.

Compound	Conversion (%) *	LCMS		^1^H NMR Chemical Shift (δ, ppm) **						
		Fragment	*m*/*z*	A (CH_2_)	B (CH_2_)	C (CH_2_)	Allylic	Olefinic	Bis-Allylic	D (CH_3_)
Hydroxytyrosyl decanoate	85.61 ± 2.9 ^e^	[M + H]^+^	309.42	2.29	1.62	1.28	-	-	-	0.90
Hydroxytyrosyl laurate	81.61 ± 1.6 ^d^	[M + H]^+^	337.48	2.29	1.63	1.29	-	-	-	0.90
Hydroxytyrosyl myristate	76.72 ± 2.7 ^c^	[M + H]^+^	365.53	2.32	1.63	1.29	-	-	-	0.90
Hydroxytyrosyl palmitate	75.13 ± 3.9 ^c^	[M + H]^+^	393.58	2.31	1.64	1.28	-	-	-	0.93
Hydroxytyrosyl stearate	61.82 ± 3.7 ^b^	[M + H]^+^	421.64	2.33	1.66	1.28	-	-	-	0.96
Hydroxytyrosyl oleate	45.91 ± 0.5 ^a^	[M + H]^+^	419.62	2.35	1.65	1.30	2.05	5.36	-	0.97
Hydroxytyrosyl eicosapentaenoate	68.13 ± 3.5 ^c^	[M + H]^+^	439.28	2.34	1.75	1.29	2.09	5.37	2.87	0.99

* Values are mean ± standard deviation (SD) for triplicate replications. Mean values with different superscript letters in the same column are significantly different (*p* < 0.05) as determined by Tukey–Kramer honestly significant difference (HSD). ** A, B, C and D represent *α*-Carboxyl, *β*-Carboxyl, aliphatic and terminal methyl group protons, respectively.

**Table 2:** Antioxidant activity of conjugates by DPPH EC_50_, ABTS ABTS (2,2-Azino-bis-3-ethylbenzothiazoline-6-sulphonic) EC_50_ and *β*-carotene bleaching tests.

Test Compound	DPPH EC_50_ (μM)	ABTS EC_50_ (μM)	*β*-Carotene Inhibition Rate (%)
Hydroxytyrosyl decanoate	6.37 ± 0.82 ^a^	2.02 ± 0.41 ^a^	57.09 ± 3.10 ^b^
Hydroxytyrosyl laurate	6.55 ± 0.54 ^a^	2.12 ± 0.22 ^a^	59.99 ± 3.08 ^c^
Hydroxytyrosyl myristate	6.42 ± 0.26 ^a^	2.22 ± 0.16 ^a^	61.73 ± 1.60 ^c^
Hydroxytyrosyl palmitate	6.71 ± 0.42 ^a^	2.19 ± 0.25 ^a^	61.45 ± 2.79 ^c^
Hydroxytyrosyl stearate	7.04 ± 0.33 ^a^	2.64 ± 0.55 ^a^	61.15 ± 2.38 ^c^
Hydroxytyrosyl oleate	6.85 ± 0.61 ^a^	2.05 ± 0.12 ^a^	62.39 ± 2.10 ^c^
Hydroxytyrosyl eicosapentaenoate	6.65 ± 0.25 ^a^	2.85 ± 0.28 ^a^	72.21 ± 3.07 ^d^
Hydroxytyrosol	6.06 ± 0.31 ^a^	2.45 ± 0.15 ^a^	45.80 ± 2.81 ^a^
BHT	6.13 ± 0.62 ^a^	3.88 ± 0.38 ^b^	56.09 ± 3.65 ^b^
*α*-tocopherol	9.87 ± 1.03 ^b^	5.82 ± 0.65 ^c^	62.43 ± 1.10 ^c^

Values are mean ± SD for triplicate replications. Mean values with different superscript letters in the same column are significantly different (*p* < 0.05) as determined by Tukey–Kramer HSD.
